# “Missing head sign”

**DOI:** 10.11604/pamj.2017.28.300.13384

**Published:** 2017-12-08

**Authors:** Deepak Gautam, Rajesh Malhotra

**Affiliations:** 1 Wellcome Trust DBT/India Alliance Fellow; 2 Department of Orthopedics, All India Institute of Medical Sciences, New Delhi 110029, India

**Keywords:** Femoral head, missing, tuberculosis of hip

## Image in medicine

A 56 years old male, farmer by occupation, presented with an altered gait. Five years back he had pain in right hip associated with fever and loss of body weight. On investigation, Erythrocyte Sedimentation Rate was raised (70 mm in first hour), X-ray revelaed dimunition of joint space and erosion of articular margins (A). On further investigation he was found to be mantoux test positive. Based on the clinicoradiological finding, he was started on Antitubercular Therapy (ATT). He was well counselled regarding the duration of treatment and the need of regular intake of drugs as prescribed from a nearby Directly Observed Treatment Short Course (DOTS) centre. After starting ATT, the fever subsided. The pain gradually settled. However, still he was having painless limp. He took ATT for 18 months as per our protocol. Two years later he followed up. X-ray revealed loss of major part of femoral head (B). The treatment options were discussed with the patient. As he was not having any problem in doing his activities of daily living, he did not opt for any surgical intervention rather went with a shoe raise option. A year further when he followed up, the X-rays revealed near complete loss of femoral head (C). At latest follow up, his x-rays showed that the femoral head was completely missing (D). We have given an acronym to this unusual presentation as a “Missing Head Sign”. As the patient was still managing his activities of daily living despite the limp, we have just kept him in regular follow up.

**Figure 1:**
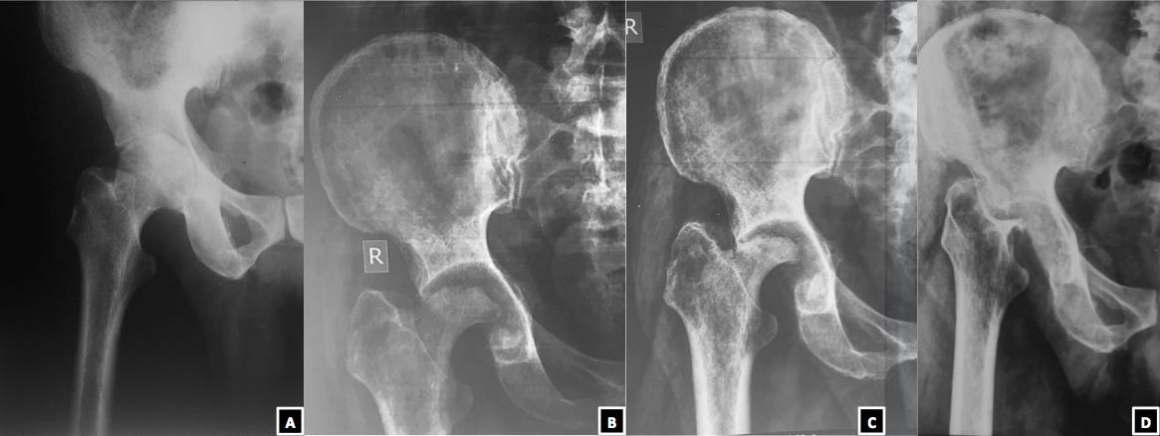
Serial x-rays of the right hip of the patient in Anteroposterior view showing complete resolution of the femoral head at the latest follow up

